# Tumour markers in breast carcinoma correlate with grade rather than with invasiveness

**DOI:** 10.1054/bjoc.2001.1995

**Published:** 2001-09

**Authors:** F Wärnberg, H Nordgren, L Bergkvist, L Holmberg

**Affiliations:** Departments of Surgery, Pathology, University Hospital Uppsala, Uppsala, S-751 85, Sweden; Department of Surgery, Central Hospital, Västerås, S-721 89, Sweden; Regional Oncological Center Uppsala-Örebro, Klostergatan 10, Uppsala, S-751 85, Sweden

**Keywords:** breast cancer, invasive, in situ, progression

## Abstract

Ductal breast carcinoma in situ (DCIS) is regarded as a precursor to invasive breast cancer. The progression from in situ to invasive cancer is however little understood. We compared some tumour markers in invasive and in situ breast carcinomas trying to find steps in this progression. We designed a semi-experimental setting and compared histopathological grading and tumour marker expression in pure DCIS (*n* = 194), small invasive lesions (*n* = 127) and lesions with both an invasive and in situ component (*n* = 305). Grading was done according to the Elston–Ellis and EORTC classification systems, respectively. Immunohistochemical staining was conducted for p53, c-erbB-2, Ki-67, ER, PR, bcl-2 and angiogenesis. All markers correlated with grade rather than with invasiveness. No marker was clearly associated with the progression from in situ to invasiveness. The expression of tumour markers was almost identical in the 2 components of mixed lesions. DCIS as a group showed a more ‘malignant picture' than invasive cancer according to the markers, probably, due to a higher proportion of poorly differentiated lesions. The step between in situ and invasive cancer seems to occur independently of tumour grade. The results suggest that well-differentiated DCIS progress to well-differentiated invasive cancer and poorly differentiated DCIS progress to poorly differentiated invasive cancer. © 2001 Cancer Research Campaign  http://www.bjcancer.com


*British Journal of Cancer* (2001) **85**(6), 869–874
© 2001 Cancer Research Campaign
doi: 10.1054/ bjoc.2001.1995, available online at http://www.idealibrary.com on IDEAL

http://www.bjcancer.com

### Tumour markers in breast carcinoma correlate with grade rather than with invasiveness

**F Wärnberg[1], H Nordgren[2], L Bergkvist[3] and L Holmberg[4]**

Departments of Surgery[1] and Pathology[2], University Hospital Uppsala, S-751 85 Uppsala, Sweden, Department of Surgery[3]; Central Hospital, S-721 89 Västerås, Sweden, Regional Oncological Center[4] Uppsala-Örebro, Klostergatan 10, S-751 85 Uppsala, Sweden

**Summary** Ductal breast carcinoma in situ (DCIS) is regarded as a precursor to invasive breast cancer. The progression from in situ to invasive cancer is however little understood. We compared some tumour markers in invasive and in situ breast carcinomas trying to find steps in this progression. We designed a semi-experimental setting and compared histopathological grading and tumour marker expression in pure DCIS ($n$ = 194), small invasive lesions ($n$ = 127) and lesions with both an invasive and in situ component ($n$ = 305). Grading was done according to the Elston–Ellis and EORTC classification systems, respectively. Immunohistochemical staining was conducted for p53, c-erbB-2, Ki-67, ER, PR, bcl-2 and angiogenesis. All markers correlated with grade rather than with invasiveness. No marker was clearly associated with the progression from in situ to invasiveness. The expression of tumour markers was almost identical in the 2 components of mixed lesions. DCIS as a group showed a more 'malignant picture' than invasive cancer according to the markers, probably, due to a higher proportion of poorly differentiated lesions. The step between in situ and invasive cancer seems to occur independently of tumour grade. The results suggest that well-differentiated DCIS progress to well-differentiated invasive cancer and poorly differentiated DCIS progress to poorly differentiated invasive cancer. © 2001 Cancer Research Campaign http://www.bjcancer.com

**Keywords**: breast cancer; invasive; in situ; progression

Ductal carcinoma in situ of the breast (DCIS) is viewed as a precursor of invasive breast cancer. Approximately half of the local recurrences after treatment for a primary DCIS are invasive (Rosen et al, 1980; Page et al, 1982; Bradley et al, 1990; Price et al, 1990; Graham et al, 1991; Swain, 1992; Fisher et al, 1993; Solin et al, 1996). Autopsy studies, however, strongly suggest that not all DCIS lesions progress to invasiveness during a patient's lifetime (Andersen et al, 1985). It would be relevant to be cognizant of which lesions have a high risk of progression. The genetic steps necessary for malignant transformation are not known and we do not know if these are reflected in the presence or absence of prognostic tumour markers. A substantial proportion of small invasive carcinomas does not have detectable areas of in situ carcinoma and possibly, in these, some crucial genetic changes have occurred early in the process. Thus, it is likely that invasive breast cancer can also occur de novo.

We aimed to determine if the distribution of some commonly used prognostic markers could help us to distinguish a group of DCIS patients with a high probability of progression to invasiveness, or if any change in tumour markers between DCIS and invasive lesions could yield interesting clues as to tumour biology. We also studied the distribution of tumour markers relative to histopathological grade. Several studies have indicated that poorly differentiated DCIS might be a precursor to poorly differentiated invasive breast cancer and well-differentiated DCIS to well-differentiated invasive breast cancer. Both histopathological grade and DNA contents have been reported to be similar in the invasive and in the in situ parts of mixed breast carcinomas (Iglehart et al, 1995; Douglas-Jones et al, 1996; Gupta et al, 1997; Buerger et al, 1999).

We studied the distribution of tumour markers in 3 cohorts of women with breast carcinoma: pure DCIS, small invasive breast carcinomas ≤ 10 mm and breast carcinomas that contained both an in situ and an invasive element. We hypothesized that tumours with both DCIS and an invasive component constituted a group in which malignant transformation was underway. Further, we assumed that small invasive carcinomas ≤10 mm without any in situ component were a group in which only a small proportion of the tumours had gone through a stage of DCIS before becoming invasive. Women with pure DCIS served as a reference group in which the crucial malignant transformation to invasiveness was assumed not to have taken place.

### MATERIALS AND METHODS

### Patients

We investigated 3 cohorts of women. 2 of the cohorts were recruited from the catchment areas of Uppsala and Västerås hospitals during the years 1986 to 1994. The first cohort contained all DCIS reported to the Swedish Cancer Registry (SCR) from these hospitals. This area is served by public health care and with no other pathological departments than the 2 in Uppsala and Västerås. 75% of the DCIS ($n$ = 145) were detected by mammography screening 2 were detected 'en passent' while operated for other reasons and the rest were detected clinically. After re-evaluation of all histopathological slides, the cohort contained 194 women with primary DCIS without any signs of invasive breast cancer.

*Received 16 February 2001*
*Revised 30 May 2001*
*Accepted 11 June 2001*

*Correspondence to:* F Wärnberg

The second cohort contained all women with an invasive breast carcinoma with a diameter of 10 mm or less as stated by the original pathological report, reported to the SCR from the same region. In 12 cases, we did not find the original paraffin-embedded material or tumour material to perform immunohistochemical (IH) staining. After re-evaluation of the slides, the cohort contained 293 women who had lesions with an invasive breast carcinoma ≤ 10 mm without (*n* = 127) or with (*n* = 166) a DCIS component. The latter group was included in the third cohort described below.

The third cohort contained all women with an invasive breast cancer diagnosed in Uppsala County between the years 1986 and 1995, where it was stated in the original pathological report that the lesion had an in situ component. All cases were re-evaluated and those with both a DCIS and an invasive breast carcinoma in the same lesion were included. The mixed lesions with a diameter of 10 mm or less during the period 1986 to 1994 were already re-evaluated and recruited from the second cohort described above. In 14 cases, we did not find any tumour material to make new slides.

Consequently, the final studied cohorts consisted of the following cases: pure DCIS (*n* = 194), invasive cancer ≤ 10 mm without an in situ component (*n* = 127) and lesions with both an invasive and an in situ component (*n* = 305).

### Methods

First, all histopathological specimens, routinely stained according to van Gieson, were re-evaluated. A DCIS lesion was classified according to the European Organisation for Research and Treatment of Cancer (EORTC) classification system (Holland et al, 1994). In this study we denoted the grades A–C (corresponding to grades I–III) to make clear that in situ and invasive lesions were classified based on different systems. If there was a dominating histopathological pattern and only a small focus of another pattern, we classified the lesion in accordance with the dominating pattern. Otherwise, we classified the lesion based on the lowest differentiated pattern in the lesions with a mixed DCIS pattern. We defined a ‘small’ focus as being at maximum 25% of the total tumour area.

Invasive breast cancer was classified based on the Elston–Ellis classification system, grades I–III (Elston and Ellis, 1991), and, in lesions with both an invasive and an in situ element, the classification was done in both parts separately according to the Elston–Ellis or the EORTC classification system, respectively. The extent of the in situ component was measured as more or less than 25% of the diameter of the invasive cancer. Even if the invasive part of a mixed lesion was very small (< 2 mm in diameter), it was referred to as an invasive breast cancer. However, in accordance with the Elston–Ellis system, no classification was possible in these small invasive lesions.

Second, new slides from the same tumour area that was histopathologically studied were prepared for IH staining of paraffin-embedded material for each tumour marker separately. The results of the staining were analysed in the in situ and the invasive components separately in all lesions with a mixed pattern. Overexpression of the p53 protein was classified into 4 groups according to the total percentage of positive tumour cells (0, 1–30, 31–60 and >60%). A cut-off limit for positive staining was chosen at p53 > 0%, regardless of the intensity of the staining. We assessed the membrane staining of the protein coded by the c-erbB-2 oncogene in the same way, but it was classified into 5 different groups (0, 1–10, 11–30, 31–60 and >60%). The staining for c-erbB-2 was considered positive when more than 30% of the cells had a moderate or strong intensity of the membrane staining or when more than 60% of the cell membranes were stained, regardless of the intensity of the staining. The intensity of the staining was classified subjectively as mild, moderate or strong. Cell proliferation was measured with reference to the percentage of tumour cells that stained for the nuclear protein Ki-67. We classified it into 5 different groups in the same way as we did with the c-erbB-2 protein. A cut-off limit for positive staining was chosen Ki-67 ≥ 10%, regardless of the intensity of the staining.

We also stained for the hormone receptors for oestrogen (ER) and progesterone (PR) and the protein coded by the *bcl-2* gene; we classified these slides based on the proportion of tumour cells stained (0–100%). We also classified the intensity of the staining as mild, moderate or strong for all different factors. Cut-off limits for positive staining was chosen ER ≥ 10%, PR ≥ 10% and *bcl-2* ≥ 10%, irrespective of the intensity of the staining.

The antibody CD 31 (or JC 70) that stains for endothelial cells was used to study angiogenesis. We used a grading system for invasive breast cancer, where we graded the number of microvessels into 3 categories: low/normal, medium and high compared with the number of microvessels in the normal parenchyma. It was a subjective evaluation performed with a light microscope in a 200 × field by our breast pathologist (HN). No hot spots were used and we looked both at the tumour and at the surrounding tissue. In the pure DCIS lesions and in the in situ part of the mixed lesions the angiogenesis was graded as normal or high in the stroma between the ducts. The amount of vessels in the stroma between normal breast ducts was used as a reference in each case. We also noted if there was a dense rim of microvessels adjacent to the basement membrane of DCIS ducts (cuffing phenomenon) as earlier described by Guidi and by Engels (Guidi et al, 1994; Engels et al, 1997). The cuffing phenomenon was scored as present (defined as microvessels completely surrounding one involved duct or surrounding at least 50% of 2 or more involved ducts) or absent.

The antibodies used were p53 clone DO-7, DAKO, c-erbB-2 poly rabbit, DAKO, Ki-67 MIB-1, Immunotech, KEBO, ER 6F11, Novocastra, PR 1A6, Novocastra, Bcl-2 clone 124, DAKO and CD31 clone JC/70A, DAKO. The staining was performed in an automatic staining machine (Ventana Medical Systems, Inc).

This study was approved by the Ethics Committee at Uppsala University Hospital.

### Statistical methods

For comparison of proportions between the groups, the chi-square test was used and differences between 2 means were assessed with Student’s *t*-test for unpaired data. Statistically significant differences were assumed when $P < 0.05$. The association between the staining of the different tumour markers in the DCIS and the invasive components of mixed lesions was described as the proportion of lesions in which both parts stained positive or both parts stained negative. The statistical trend of staining by histopathological tumour grades A–C or I–III for the different tumour markers in invasive and in situ carcinomas, respectively, was calculated with logistic regression models that used grade as an ordinal variable and estimated odd ratios with 95% confidence interval. The Microsoft Statistica and SAS software were used for all statistical calculations.

© 2001 Cancer Research Campaign

### RESULTS

Among the 626 women included in this study we could not find paraffin-embedded material in 30 cases (5%). These women were included in analyses using the van Gieson staining technique only. In another 46 (7%) cases one or more of the IH staining was not performed, mainly due to a small tumour size that did not allow us to prepare all slides needed.

The mean age in the different cohorts was 60.0 years in women with pure DCIS, 60.0 years in women with invasive breast carcinoma ≤10 mm without an in situ component and 59.0 years in women with lesions with both an invasive and an in situ component. When we calculated the mean size of the lesions in the different cohorts, we only included lesions described in mm in the original pathological report. The mean size was 14.9 mm in women with pure DCIS ($n = 120$), 8.0 mm in women with invasive breast carcinoma ≤10 mm without an in situ component ($n = 127$). In the cohort with mixed lesions the subgroup of lesions ≤10 mm had a mean size of 7.8 mm ($n = 172$) and the subgroup with larger lesions had a mean size of 23.9 mm ($n = 90$). In the group with mixed lesions only the invasive component of the lesion was measured, and the in situ part was described as more or less than 25% of the invasive component.

The proportions of lesions showing positive staining for the different tumour markers and the distribution of lesions according to histopathological grade in the different cohorts are summarised in Table 1. Overexpression of both p53 and c-erbB-2 protein was more frequently seen in the pure DCIS lesions than in the invasive lesions, both with and without an in situ component. The difference was statistically significant when we compared DCIS with all invasive lesions together (P53, $P < 0.001$ and c-erbB-2, $P < 0.05$). Proliferation, measured with Ki-67 staining, was less frequent in the pure DCIS group, but this difference was not statistically significant. Although the proportions of positive-stained tumour cells for ER, PR and *bcl-2* were lower in pure DCIS compared with the invasive lesions, the difference failed to reach statistical significance. Because angiogenesis was classified in different ways, it was not possible to compare the proportion of positive staining between pure DCIS and invasive carcinomas.

The DCIS and the invasive carcinomas were graded using different histopathological systems. As shown in Table 1, in pure DCIS there were few well-differentiated lesions (i.e., grade A) and a large proportion of poorly differentiated lesions (i.e., grade C). In the small invasive tumours, without an in situ component, about 50% were grade I while only 4.9% were grade III. In the mixed lesions over 50% were grade I and 8.3% were grade III. In the cohort with mixed lesions the larger tumours (> 10 mm) were more often poorly differentiated (grade III) than the smaller tumours ($P < 0.001$).

An unexpected finding was the more ‘malignant picture’ in the DCIS cohort compared with the invasive tumours. Because the distribution of grade was materially different in the cohorts and because grade might be a stronger marker for tumour progression in comparison with any of the other tumour markers we stained for, we analysed the staining for the tumour markers by grade. These results are presented in Table 2.

Based on tumour grade, almost every biological event that the different staining represented had a statistically significant trend of a more ‘malignant picture’ in both the in situ and the invasive cohorts (Table 2). The frequency of p53 and c-erbB-2 was highest in grade C and grade III lesions in all cohorts. Further, proliferation was more pronounced in poorly differentiated lesions. A higher proliferation was seen in the invasive cohorts than in pure DCIS when we compared each grade separately (A vs. I, B vs. II and C vs. III). This difference was statistically significant between grade B and grade II (9% vs. 34%, $P < 0.001$) and between grade C and grade III (33% vs. 56%, $P < 0.05$).

Poorly differentiated lesions were found to stain less for hormone receptors and *bcl-2* than well-differentiated tumours in all cohorts. When we compared each grade individually, the pure DCIS lesions exhibited a higher percentage of staining than the invasive lesions (A vs. I, B vs. II and C vs. III). The poorly differentiated lesions displayed the most pronounced angiogenesis in all cohorts. Moreover, in pure DCIS lesions the cuffing phenomenon of microvessels was more common in grade C lesions as compared with grades A and B lesions ($P < 0.01$) (Table 2).

We looked at the in situ component of the mixed lesions and the same pattern of a more ‘malignant picture’ was noted according to grade (Table 2). The correlation between the DCIS component and the invasive part of the mixed lesions, based on the staining for the different tumour markers, is displayed in Table 3. The staining of the invasive and in situ components was highly correlated for all

**Table 1** The percentage of women indicating positive staining for different tumour markers and the percentage and number of women according to histopathological grade by subgroup. DCIS was classified in accordance with the EORTC system (grades A–C) and invasive breast carcinoma according to the Elston–Ellis system (grades I–III). Cut-off limits are given in the text

| | DCIS pure ($n = 194$) | Invasive breast carcinoma ≤10 mm without an in situ component ($n = 127$) | Invasive breast carcinoma with an in situ component ($n = 305$) |
|---|---|---|---|
| P53 | 40% | 25% | 28% |
| C-erbB-2 | 55% | 41% | 49% |
| Ki 67 | 19% | 21% | 27% |
| ER + | 68% | 78% | 71% |
| PR + | 43% | 52% | 52% |
| Bcl2+ | 48% | 52% | 57% |
| CD31 | | | |
| Stroma | 35% | 27% | 35% |
| Cuffing | 23% | – | – |
| Grade A-I | 6.7% (13) | 51.2% (63) | 56.3% (156) |
| Grade B-II | 50.5% (98) | 43.9% (54) | 35.4% (98) |
| Grade C-III | 42.8% (83) | 4.9% (6) | 8.3% (23) |

*© 2001 Cancer Research Campaign* 

**Table 2** The percentage of women with a positive staining for different tumour markers as a function of subgroup and grade. The number and percentage of women according to histopathological grade are given for the different parts in the mixed lesions. DCIS was classified according to the EORTC system (grades A–C) and invasive breast carcinoma according to the Elston–Ellis system (grades I–III). Cut-off limits are given in the text

| | DCIS pure | | | Invasive breast carcinoma ≤10 mm without an in situ component | | | Invasive breast carcinoma (I–III) with an in situ component (A–C) | | | | | |
|---|---|---|---|---|---|---|---|---|---|---|---|---|
| **Grade** | **A** | **B** | **C** | **I** | **II** | **III** | **I** 56.3% (*n* = 156) | **II** 35.4% (*n* = 98) | **III** 8.3% (*n* = 23) | **A** 18.1% (*n* = 49) | **B** 51.3% (*n* = 139) | **C** 30.6% (*n* = 83) |
| P53 | 0% | 25% | 63%* | 13% | 21% | 67%* | 17% | 33% | 71%* | 4% | 15% | 47%* |
| C-erbB-2 | 31% | 27% | 75%* | 35% | 33% | 83% | 43% | 53% | 70%* | 30% | 49% | 73%* |
| Ki 67 | 0% | 9% | 33%* | 5% | 23% | 67%* | 15% | 41% | 52%* | 12% | 22% | 46%* |
| ER + | 92% | 85% | 46%* | 81% | 71% | 17%* | 77% | 70% | 30%* | 81% | 78% | 57%* |
| PR + | 83% | 54% | 25%* | 46% | 46% | 0% | 57% | 56% | 21%* | 55% | 69% | 41% |
| BCl2+ | 83% | 54% | 37%* | 54% | 41% | 33% | 66% | 48% | 37%* | 70% | 65% | 40%* |
| CD31 | | | | | | | | | | | | |
| Stroma | 25% | 22% | 52%* | 13% | 40% | 100%* | 25% | 42% | 68% | 13% | 29% | 56%* |
| Cuffing | 8% | 12% | 31%* | – | – | – | – | – | – | 0% | 6% | 21%* |

*Statistically significant trend of the staining for the tumour markers according to grade in the different groups.

**Table 3** The correlation of the staining of different tumour markers between the invasive and the in situ part in mixed breast carcinomas. The percentage of lesions with either both parts positive or both parts negative (++/––) are given. The numbers of lesions, invasive part and in situ part respectively, with positive (+), negative (–) staining or where staining was not performed (np) are given. Cut-off limits are given in the text

| | **P53** | **C-erb-B2** | **Ki 67** | **ER** | **PR** | **Bcl2** | **CD 31 stroma** |
|---|---|---|---|---|---|---|---|
| Invasive breast carcinoma ≤ 10 mm with in situ, ++/–– | 80% | 93% | 89% | 91% | 82% | 91% | 75% |
| Invasive part + / – / np | 46/115/15 | 74/90/12 | 41/123/12 | 121/26/29 | 78/66/32 | 82/66/28 | 39/119/18 |
| In situ part + / – / np | 26/127/23 | 80/87/9 | 40/118/18 | 120/22/34 | 86/51/39 | 79/63/34 | 45/103/28 |
| Invasive breast carcinoma > 10 mm with in situ ++ / –– | 93% | 93% | 91% | 96% | 87% | 86% | 73% |
| Invasive part + / – / np | 30/84/15 | 63/55/11 | 35/83/11 | 63/50/16 | 54/57/18 | 67/47/15 | 53/55/21 |
| In situ part + / – / np | 34/79/16 | 67/52/10 | 34/84/11 | 67/47/15 | 59/53/17 | 68/46/15 | 46/66/17 |

**Table 4** The correlation between the in situ and the invasive parts of mixed lesions based on the histopathological grade. Numbers are given. DCIS was classified according to the EORTC system (grades A–C) and invasive breast carcinoma according to the Elston–Ellis system (grades I–III)

| **Lesions with both a DCIS and an invasive component** | **Invasive component** | | |
|---|---|---|---|
| | **Grade I** | **Grade II** | **Grade III** |
| DCIS component | | | |
| Grade A | 40 | 5 | 0 |
| Grade B | 94 | 39 | 1 |
| Grade C | 10 | 47 | 23 |

tumour markers in both small and large mixed tumours. The distribution of histopathological grade in the in situ component of the mixed lesions was more like the distribution of histopathological grade in the lesions with pure DCIS than in the corresponding invasive components.

The correlation between the histopathological grade in the DCIS and the invasive components of mixed lesions is depicted in Table 4. In 10 of the 144 invasive tumours (7%), classified as grade I, the DCIS component was classified as grade C. Only one of the 24 invasive tumours (4%), classified as grade III, was correlated with a grade A or grade B DCIS.

We also studied the pattern of the tumour markers in all mixed lesions based on whether the extent of the in situ component was greater or smaller than 25% of the diameter of the corresponding invasive component. No statistically significant differences were seen between these 2 patterns.

### DISCUSSION

We constructed a semi-experimental setting to observe whether there were any marked differences in the distribution of tumour markers between in situ and invasive lesions. We could not detect any pattern that signalled the progression from an in situ stage to invasiveness. Instead, the main finding was that the expression of the different tumour markers strongly correlated with tumour grade in both DCIS and invasive breast cancer.

We studied a large number of tumours in well-defined subgroups. A high proportion of tumours could be IH stained with few losses because of missing tumour material. We do not know how different fixation and storage of the tumours may have influenced the results. However, we recruited the tumours from the same pathological departments and from the same time period and we have no reason to believe that the technical difficulties would be different between in situ and invasive components. Thus, comparisons between the cohorts should be valid though any

*© 2001 Cancer Research Campaign*

non-differential misclassification may lower the power to detect small or moderate differences.

The cohort with pure DCIS showed a more 'malignant pattern' regarding all tumour markers, except for proliferation. Similar to other studies, the overexpression of c-erb-B2 in DCIS was most frequently seen in poorly differentiated lesions. C-erbB-2 overexpression was also more frequently found in the cohort with pure DCIS compared with invasive lesions (Allred et al, 1992; Somerville et al, 1992; Leal et al, 1995), but if we compared each grade separately it was more common in invasive carcinomas (A/I, B/II and C/III). Contrary to Allred's (Allred et al, 1992) suggestion, the present results do not support the hypothesis that overexpression of c-erbB-2 decreases within individual tumours as they evolve from in situ to invasive carcinomas. As in our study, Igelhart et al found that the expression of c-erbB-2 was similar in both growth phases of lesions with an invasive and an in situ component (Iglehart et al, 1995).

The quantification of angiogenesis in breast cancer is a prevailing topic (Fox et al, 1995) and there is no standard method used for both DCIS and invasive breast cancer. In this study we used a semi-quantitative method and then classified the amount of microvessels as high or low. The angiogenesis in DCIS and invasive carcinomas was not classified in exactly the same way. However, we did compare the angiogenesis by grade in the different cohorts separately. When we compared the angiogenesis in the 2 growth stages of the mixed lesions, DCIS and invasive carcinoma, we found a statistically significant correlation between the components though not as strong as among the other tumour markers.

We used 2 histopathological classification systems for DCIS and invasive carcinomas since there is no single system dealing with both entities. In the mixed lesions the grade of the 2 components, in situ and invasive carcinoma, was basically but not totally corresponding (Table 4). When we studied the expression of the different tumour markers, however, the expression of the markers in the 2 components was almost identical (Table 3). These findings support the theory that a well-differentiated DCIS progresses to a well-differentiated invasive cancer and a poorly differentiated DCIS progresses to a poorly differentiated invasive cancer. Consequently, it is probable that the step between invasive and in situ carcinoma occurs independently of tumour grade.

The distribution of lesions by grade in pure DCIS and small invasive lesions (≤10 mm) (Table 1) showed a small proportion of well-differentiated DCIS and, on the contrary, a large proportion of well-differentiated small invasive carcinomas. One explanation of the different distributions of pure DCIS by grade compared with small invasive carcinomas can be that poorly differentiated DCIS with calcifications is more easily detected by mammography than DCIS without calcifications (Stomper et al, 1989). 75% of the DCIS were screening detected in this study. However, the difference in distribution by grade between pure DCIS and small invasive lesions may also be artificial in that we used different histopathological classification systems in the 2 groups. The DCIS and the invasive parts of mixed lesions appeared very similar regarding the staining of all tumour markers. However, if we classified the DCIS component of the mixed lesions based on the EORTC classification system and looked at the pattern of the staining for the tumour markers by grades A–C, the pattern shifted towards the pattern of the cohort with pure DCIS (Table 2). Furthermore, the distribution by grade was more comparable to that of the cohort with pure DCIS. This distribution pattern may indicate that histopathological grades A–C and grades I–III are not exchangeable or do not represent the same level of biological differentiation.

Grade is prognostically important within the in situ and invasive groups. The other biological markers we studied – reflecting more of singular entities than grade – are all strongly correlated with grade. In in situ carcinomas high grade entails a higher risk of local new events (of which 50% are invasive); in invasive carcinomas high grade is also associated with risk of distant metastases. It is intriguing that patterns of grade or the other markers did not seem to mark the transition from in situ to invasive carcinoma, a step believed to be crucial for the tumour's ability to metastasise. We postulate that factors that signal the tumour's ability to invade may be powerful prognostic factors and that they may be searched for in a setting comparable to ours.

### ACKNOWLEDGEMENTS

This study was supported by grants from the Swedish Cancer Society, Västmanlands research fund against cancer and the Lion's cancer fund.

### REFERENCES

Allred DC et al (1992) Overexpression of HER-2/neu and its relationship with other prognostic factors change during the progression of in situ to invasive breast cancer. *Hum Pathol* **23**: 974–979

Andersen J, Nielsen M and Christensen L (1985) New aspects of the natural history of in situ and invasive carcinoma in the female breast. Results from autopsy investigations. *Verh Dtsch Ges Path* **69**: 88–95

Bradley SJ, Weaver DW and Bouwman DL et al (1990) Alternatives in the surgical management of in situ breast cancer. A meta-analysis of outcome. *Am Surgeon* **56**: 428–432

Buerger H et al (1999) Comparative genomic hybridization of ductal carcinoma in situ of the breast – evidence of multiple genetic pathways. *J Pathol* **187**: 396–402

Douglas-Jones AG et al (1996) A critical appraisal of six modern classifications of ductal carcinoma in situ of the breast (DCIS): correlation with grade of associated invasive carcinoma. *Histopathol* **29**: 397–409

Elston CW and Ellis IO et al (1991) Pathological prognostic factors in breast cancer I. The value of histological grade in breast cancer: experience from a large study with long term follow up. *Histopathol* **19**: 403–410

Engels K et al (1997) Distinct angiogenic patterns are associated with high-grade in situ ductal carcinomas of the breast. *J Pathol* **181**: 207–212

Fisher B et al (1993) Lumpectomy compared with lumpectomy and radiation therapy for the treatment of intraductal breast cancer. *N Engl J Med* **328**: 1581–1586

Fox SB et al (1995) Quantitation and prognostic value of breast cancer angiogenesis: comparison of microvessel density, Chalkley count, and computer image analysis. *J Pathol* **177**: 275–283

Graham MD, Lakhani S and Gazet JC (1991) Breast conserving surgery in the management of in situ breast carcinoma. *Eur J Surg Oncol* **17**: 258–264

Guidi AJ et al (1994) Microvessel density and distribution in ductal carcinoma in situ of the breast. *J Nat Cancer Inst* **86**: 614–619

Gupta SK et al (1997) The clinical of breast carcinoma is probably determined at the preinvasive stage (ductal carcinoma in situ). *Cancer* **80**: 1740–1745

Holland R et al (1994) Ductal carcinoma in situ: A proposal for a new classification. *Sem Diag Pathol* **11**: 167–180

Iglehart JD et al (1995) Maintenance of DNA content and erbB-2 alterations in intraductal and invasive phases of mammary cancer. *Breast Cancer Res Treat* **34**: 253–263

Leal CB et al (1995) Ductal carcinoma in situ of the breast. Histologic categorization and its relationship to ploidy and immunohistochemical expression of hormone receptors, p53, and c-erbB-2 Protein. *Cancer* **75**: 2123–2131

Page DL et al (1982) Intraductal carcinoma of the breast: Follow-up after biopsy only. *Cancer* **49**: 751–758

Price P et al (1990) Duct carcinoma in situ: predictors of local recurrence and progression in patients treated by surgery alone. *Br J Cancer* **61**: 869–872

© 2001 Cancer Research Campaign 

Rosen PP, Braun DW and Kinne DE (1980) The clinical significance of pre-invasive breast carcinoma. *Cancer* **46**: 919–925

Solin LJ et al (1996) Fifteen-year results of breast-conserving surgery and definitive breast irradiation for the treatment of ductal carcinoma in situ of the breast. *J Clin Oncol* **14**: 754–763

Somerville JE, Clarke LA and Biggart JD (1992) C-erbB-2 overexpression and histological type of in situ and invasive breast carcinoma. *J Clin Pathol* **45**: 16–20

Stomper PC et al (1989) Clinically occult ductal carcinoma in situ detected with mammography: analysis of 100 cases with radiologic-pathologic correlation. *Radiology* **172**: 235–241

Swain SM et al (1992) Ductal carcinoma in situ. *Cancer Invest* **10**: 443–454

*© 2001 Cancer Research Campaign*
